# TB Meningitis in HIV-Positive Patients in Europe and Argentina: Clinical Outcome and Factors Associated with Mortality

**DOI:** 10.1155/2013/373601

**Published:** 2013-12-31

**Authors:** Anne Marie W. Efsen, Alexander M. Panteleev, Daniel Grint, Daria N. Podlekareva, Anna Vassilenko, Aza Rakhmanova, Indra Zeltina, Marcelo H. Losso, Robert F. Miller, Enrico Girardi, Joan Caylá, Frank A. Post, Jose M. Miro, Mathias Bruyand, Hansjakob Furrer, Niels Obel, Jens D. Lundgren, Amanda Mocroft, Ole Kirk, HIV/TB Study Group

**Affiliations:** ^1^Copenhagen HIV Programme, Rigshospitalet, Faculty of Health and Medical Sciences, Copenhagen University Hospital and University of Copenhagen, 2200 Copenhagen N, Denmark; ^2^TB Hospital No. 2, Russian Federation, 195267 Saint Petersburg, Russia; ^3^University College London, Royal Free Campus, London NW3 2PF, UK; ^4^Belorusian State Medical University, Minsk 220002, Belarus; ^5^St. Petersburg AIDS Centre, 193167 Saint Petersburg, Russia; ^6^Infectology Centre of Latvia, 1006 Riga, Latvia; ^7^Hospital JM Ramos Mejia, Servicio de Inmunocomprometidos, CP 1221 Buenos Aires, Argentina; ^8^Centre for Sexual Health & HIV Research, Mortimer Market Centre, University College London, London WC1E 6JB, UK; ^9^Istituto Nazionale Malattie Infettive L Spallanzani, 00149 Rome, Italy; ^10^Servicio de Epidemiología, Agencia de Salud Pública de Barcelona, CIBER Epidemiología y Salud Pública (CIBERESP), 08 036 Barcelona, Spain; ^11^King's College London School of Medicine, London SE5 9RS, UK; ^12^Hospital Clinic-IDIBAPS, University of Barcelona, 08 036 Barcelona, Spain; ^13^Centre Hospitalier Universitaire (CHU) de Bordeaux, COREVIH Aquitaine, 33000 Bordeaux, France; ^14^INSERM, ISPED, Centre Inserm U897-Epidemiologie-Biostatistique, 33000 Bordeaux, France; ^15^Department of Infectious Diseases, Bern University Hospital and University of Bern, CH-3010 Bern, Switzerland; ^16^Department of Infectious Diseases, Copenhagen University Hospital/Rigshospitalet, 2100 Copenhagen, Denmark

## Abstract

*Objectives.* The study aimed at describing characteristics and outcome of tuberculous meningitis (TBM) in HIV-positive patients and comparing these parameters with those of extrapulmonary TB (TBEP) and pulmonary TB (TBP). *Methods.* Kaplan-Meier estimation and Poisson regression models were used to assess the mortality following TB diagnosis and to evaluate potential prognostic factors for the 3 groups of TB patients separately. *Results.* A total of 100 patients with TBM, 601 with TBEP, and 371 TBP were included. Patients with TBM had lower CD4 cell counts and only 17.0% received antiretroviral therapy (ART) at TB diagnosis. The cumulative probability of death at 12 months following TB was 51.2% for TBM (95% CI 41.4–61.6%), 12.3% for TBP (8.9–15.7%), and 19.4% for TBEP (16.1–22.6) (*P* < 0.0001; log-rank test). For TBM, factors associated with a poorer prognosis were not being on ART (adjusted incidence rate ratio (aIRR) 4.00 (1.72–9.09), a prior AIDS diagnosis (aIRR = 4.82 (2.61–8.92)), and receiving care in Eastern Europe (aIRR = 5.41 (2.58–11.34))). *Conclusions.* TBM among HIV-positive patients was associated with a high mortality rate, especially for patients from Eastern Europe and patients with advanced HIV-infection, which urgently calls for public health interventions to improve both TB and HIV aspects of patient management.

## 1. Introduction

Tuberculous meningitis (TBM) is one of the most devastating manifestations of extrapulmonary tuberculosis (TBEP) and is associated with severe morbidity and high mortality [1]. HIV-positive individuals are known to be at increased risk of developing TBEP including TBM, particularly those patients with more advanced levels of immunodeficiency [2–4]. Further, it has been shown that TBM among HIV-positive patients in the period prior to combination antiretroviral therapy (cART) had higher mortality rates compared to HIV-negative individuals [3, 5].

Establishment of a TB diagnosis in HIV-positive patients poses a clinical challenge. This is true for both pulmonary TB (TBP) and TBEP and perhaps especially so for a TBM diagnosis [6]. Early diagnosis is important because it improves the overall prognosis; however, identification of acid-fast bacilli in the cerebrospinal fluid (CSF) and culture of *Mycobacterium tuberculosis* (*Mtb*) lacks sensitivity, and therefore a TBM diagnosis is often based on clinical suspicion combined with empirical decision making [3, 7].

No randomized controlled trials have investigated the optimal treatment regimen for TBM. Current treatment recommendations of 9–12 months of anti-TB treatment are extrapolated from clinical trials of treatment for TBP, with some guidelines advising a regimen consisting of rifampicin, isoniazid, pyrazinamide, and ethambutol [8], while others recommending that ethambutol can be replaced by either streptomycin or ethionamide [9, 10].

The overall aim of this study was to describe the patient characteristics, TB regimens, treatment outcomes, and prognostic factors for HIV-positive patients with TBM and compare these parameters to those of patients with TBEP or TBP.

## 2. Methods

### 2.1. The HIV/TB Study

The HIV/TB study is a retrospective, observational cohort study based on collaboration between fifty four clinics and cohorts in Eastern Europe (EE), Central/Northern Europe (CNE), Southern Europe (SE), and Argentina (AR). The study included patients of 16 years or older who were diagnosed with TB between January 2004 and December 2006 and who were either known to be HIV-positive or diagnosed with HIV up to 3 months after their TB diagnosis. Information was collected on standardized Case Report Forms (CRFs, available at http://www.cphiv.dk), and details of the study have previously been published [11]. In this paper, we describe characteristics and outcomes for patients with TBM, TBP, and TBEP.

Patients were categorized as having TBP if TB disease was limited to either the lungs, pleura, or both. TBEP included cases with either TB limited to at least one extrapulmonary organ system (and potentially involving lungs and pleura), including miliary TB or *Mtb* isolated from blood or bone marrow. Disseminated TB was defined as involvement of at least two extrapulmonary organ systems. Patients with TBM were categorized separately regardless of having additional pulmonary or other extrapulmonary involvement. The TBM diagnosis was established by evidence of mycobacteria in CSF (culture/AFB/PCR positive in CSF) or by empirical decision of the clinician primarily based on a combination of central nervous system symptoms (e.g., headache, altered consciousness, dizziness, and nausea) and contemporaneous documentation of extracranial TB.

Patients were categorized as having either a definite TB diagnosis (culture/PCR positive), a probable TB diagnosis (positive microscopy for acid-fast-bacilli (AFB)/granulomatous inflammation on histology), or a presumptive TB diagnosis (based on clinical suspicion, where treatment was initiated and not subsequently stopped because the TB diagnosis was ruled out) as previously reported [11].

TB disease outcomes were classified as either “success,” defined as either cure or treatment completed, or “fail,” defined as either treatment failure, treatment deferred, treatment interrupted, death, or loss to follow-up [12].

### 2.2. Statistical Methods

Descriptive statistics were used to describe patients' characteristics in the three TB disease groups. Continuous variables were described using medians and interquartile ranges, categorical variables using numbers and percentages, and compared between diagnosis groups using Mann-Whitney and *χ* ^2^-tests, respectively.

The date of TB diagnosis (baseline) was either the date when a positive TB sample was obtained or the date when TB treatment was initiated, whichever came first.

Kaplan-Meier estimation was used to describe the proportion of patients who had died following TB diagnosis, stratified by type of TB. Poisson regression models were used to evaluate potential prognostic factors for the three groups of TB patients separately, and the model included the following variables that were identified *a priori*: geographical region, age, CD4 cell count, usage of cART at time of TB, prior AIDS, certainty of the TB diagnosis, presence of disseminated TB disease, presence of multidrug resistant (MDR) TB, and usage of a rifamycin in the initial anti-TB regimen. As the focus was type of TB, interactions between type of TB and other variables were investigated to determine if the relationships differed, for example, if the relationship between age and outcome differed depending on the type of TB diagnosis, and stratified results presented.

Sensitivity analyses were performed to investigate whether consistent results were found among patients with a definitive diagnosis compared to those with a presumptive or possible diagnosis and also to determine the relationship between type of TB diagnosis and death in the first 12 months of follow-up, where mortality is known to be the worst, particularly in Eastern Europe [13].

Follow-up was until date of death or the last date when the patient was known to be alive or July 2010, whichever occurred first. All analyses were performed using SAS, version 9.3 (Statistical analysis Software, version 9.3, Cary, NC, USA).

### 2.3. Ethical Approval

The overall study protocol was approved by local ethics committees for each of the participating clinics and cohorts as per local regulations and was registered with the Danish Data Protection Agency.

## 3. Results

### 3.1. Patient Characteristics and Treatment

Of the 1072 patients with a classifiable TB diagnosis, 100 (9.3%) were diagnosed with TBM, 371 (34.6%) with TBP, and 601 (56.1%) with TBEP. HIV and TB clinical characteristics of the patients are presented in Table 1. The proportion of TB patients presenting with TBM varied between Europe and Argentina (10.4%, 10.4%, and 8.3% in Eastern, Central/Northern, and Southern Europe, respectively compared with 4.3% in Argentina, *P* = 0.072).

The majority of patients, 981 (91.5%), in all three groups were known to be HIV-positive at the time of TB diagnosis. Patients with TBM had significantly lower CD4 cell counts compared to those with TBEP and TBP (*P* < 0.0001). Significantly more patients with TBM had a prior AIDS diagnosis other than TB: 39 (40.6%) compared to 112 (34.0%) TBP and 161 (29.0%) TBEP patients (*P* = 0.043) (Table 1). A significantly higher proportion of patients with TBM had an AIDS defining illness within 3 months before or after their TB diagnosis, 33 (33.0%) compared to 49 (13.2%) patients with TBP and 89 (14.8%) with TBEP (*P* < 0.0001), possibly reflecting their more advanced disease. In this time, only 3 AIDS defining illnesses occurred in >10 patients, oesophageal candidiasis (*n* = 84), pneumocystis jiroveci pneumonia (*n* = 25), and toxoplasmosis (*n* = 12), with other AIDS defining illnesses occurring in 50 patients. Patients with TBM were more likely to have one of the other, less frequently occurring AIDS defining illnesses (15/33, 45.5%), compared to those with TBP (13/49, 26.5%) or TBEP (22/89, 24.7%, *P* = 0.078), although this was marginally statistically significant. Among the 15 other diagnoses in those with TBM, there were 4 diagnoses of AIDS dementia, 4 of HIV wasting, 3 of extraocular cytomegalovirus, and 1 each of cryptococcosis, cytomegalovirus retinitis, recurrent herpes simplex virus, and progressive multifocal leucoencephalopathy. Of those known to be HIV positive at time of TB diagnosis, 16 (16.7%) of the TBM patients were receiving cART at time of TB diagnosis compared with 74 (22.5%) and 87 (15.7%) in the TBP and TBEP groups, *P* = 0.035, and, of those with HIV-RNA measurements available at time of TB diagnosis, 7 (21.9%) in the TBM group had a viral load <400 copies/mL, compared to 32 (26.9%) of those with TBP and 27 (13.8%) in the TBEP group (*P* = 0.015) (Table 1).

The TB diagnosis was less often definite for TBM patients, 46 (46.0%), compared with 244 (65.8%) and 358 (59.6%) in the TBP and TBEP groups, *P* = 0.0037, and in 38 (38.0%) patients *Mtb* was documented in cerebrospinal fluid (CSF) by microscopy (24), PCR (7), and/or culture (17). Severity of TB disease was similar in the TBM and TBEP groups: 77 (77%) and 515 (85.7%) had disseminated TB, respectively, and as per definition, no patients in the TBP group (*P* = 0.038 between TBM and TBEP). Of the TBM and TBEP patients, 69 (69.0%) and 478 (79.5%) had TBP as well (Table 1).

Significantly more patients with TBM were classified as having MDR-TB, 5 of 21 (23.8%) TBM patients with DST available, compared to 14 of 96 (14.7%) with TBP and 8 of 139 (5.8%) with TBEP (*P* = 0.011). Significantly fewer patients with TBM were treated with an initial regimen containing isoniazid, pyrazinamide, and rifamycin (*P* = 0.016) and more patients in the TBM group received at least one 2nd line drug (*P* = 0.0007) as part of their initial regimen compared with the TBP and TBEP groups (Table 1).

### 3.2. Patient Outcome

**Figure 1:**
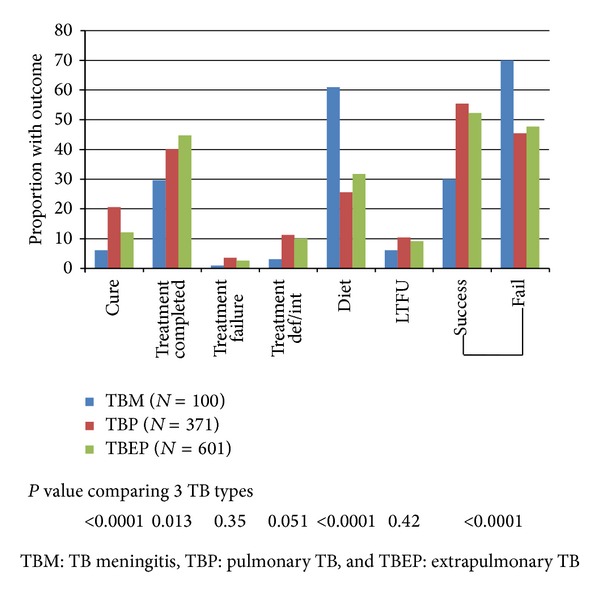
Outcomes following TB diagnosis.

**Figure 2:**
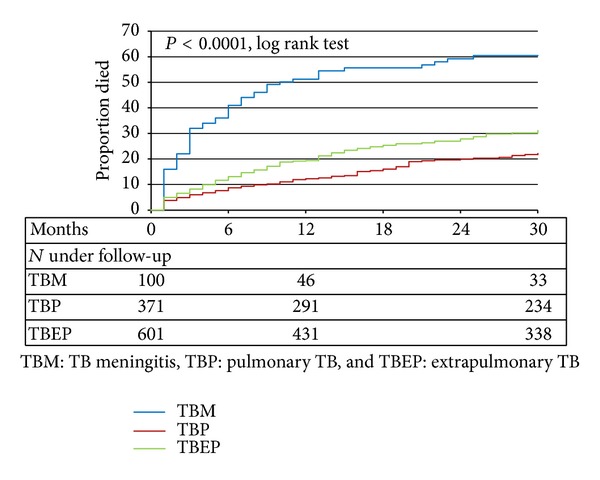
Kaplan-Meier progression to death.

Only 30 (30.0%) patients with a TBM diagnosis attained successful treatment outcome (cure or treatment completed) compared to 204 (55.2%) and 314 (52.3%) in the TBP and TBEP groups, respectively, *P* < 0.0001. Sixty-one patients in the TBM group died (61.0%), 95 in the TBP group (25.6%), and 191 in the TBEP group (31.8%), *P* < 0.0001 (Figure 1). The loss to follow-up rate during therapy was the lowest for TBM patients and overall below 10% (Figure 1). The overall mortality rate was considerably higher in the TBM group (39.3, 95% CI 29.4–49.2 per 100 person years of follow-up (PYFU)) compared to the TBEP group (13.0, 95% CI 11.2–14.7 per 100 PYFU) and the TBP group (6.4, 95% CI 5.1–7.7 per 100 PYFU). 207 deaths (59.7%) occurred within the first 12 months following TB diagnosis; this proportion was significantly higher for TBM (51, 83.6%) compared to 44 (46.3%) for TBP and 112 (58.6%) for TBEP (*P* < 0.0001). Using Kaplan-Meier estimation, the cumulative probability of death at 12 months following TB diagnosis was 51.2% of those with TBM (95% CI 41.4–61.6%), compared to 12.3% for those with TBP (95% CI 8.9–15.7%) and 19.4% for those with TBEP (95% CI 16.1–22.6) (*P* < 0.0001, log-rank test) (Figure 2).

Table 2 shows the adjusted incidence rate ratios (aIRRs) for *a priori* selected variables within each of the TB groups. Patients from Eastern Europe (compared to all other regions combined) had an approximately 5-fold increased risk of dying following TBM, an 11-fold increased risk of dying following TBEP, and a 3-fold increased risk for dying following TBP (test for interaction between geographic region and TB group, *P* = 0.0004). Prior AIDS was strongly associated with death among TBM patients, and less pronounced so among TBEP patients, but not among TBP patients. Being on cART at TB diagnosis was associated with approximately 2/3 reduction in risk of dying in all three groups, whereas low CD4 cell count at time of TB was an independent prognostic factor for patients with TBP and TBEP but not for TBM patients. TB resistance and usage of a rifamycin containing initial regimen were not associated with death in any of the groups, perhaps at least partially due to low numbers in some of the groups.

### 3.3. Sensitivity Analyses

In a sensitivity analysis confined to patients with a definitive diagnosis, consistent results were found for all diagnoses to those shown in Table 2, although the confidence intervals became very wide because of the smaller number of patients included which makes any firm conclusions about consistency difficult. For example, there were 46 TBM patients with a definitive diagnosis of TB. Patients from Eastern Europe had almost a 3-fold increased incidence of death following TBM compared to patients from all other regions compared (aIRR 2.96; 95% CI 1.09–8.06, *P* = 0.029). Being on cART at TBM was associated with a lower risk of death (aIRR 0.25; 95% CI 0.07–0.87, *P* = 0.017). An analysis further limited to the 38 patients where *Mtb* was documented from the CSF showed similar results, but with even less precision (data not shown).

A sensitivity analysis was also performed limited to the first 12 months of follow-up. As expected, as the majority of patients with TBM died within 12 months, these results were highly consistent with those shown in Table 2. The results were also highly consistent for TBP. In the first 12 months after diagnosis, there was less of a difference between regions for TBEP; after adjustment, patients from Eastern Europe had almost a 7-fold increased incidence of death compared to other regions (aIRR 6.74; 95% CI 4.38–10.36, *P* < 0.0001). The other results were consistent with those shown in Table 2.

## 4. Discussion

The present study demonstrates that among HIV-positive patients in Europe and Argentina, TBM is associated with a high mortality rate, which is significantly higher than among patients with either TBP or TBEP without meningeal involvement. Approximately 51% of patients with TBM died within 12 months of TB diagnosis, and our overall findings are consistent with those from other continents [10]. A randomized, controlled trial from Vietnam found that among 96 TBM patients coinfected with HIV, approximately 65% died within 9 months after anti-TB treatment had been initiated [3]. A retrospective study from Argentina found a mortality rate of 63% during hospitalization among 101 TBM patients coinfected with HIV [6], while a study from Zimbabwe documented 67% in-hospital mortality among 21 culture positive TBM patients coinfected with HIV [14].

Patients included in the previous studies were characterized by severe immune suppression at the time of TBM diagnosis and none had received cART. In the present study, only 17% of TBM patients were on cART at TBM diagnosis, and the majority of patients with TBM were immunosuppressed. Factors which were significantly associated with death following TBM included region of follow-up (Eastern Europe), having a prior (non-TB) AIDS diagnosis, and not receiving cART.

Only 30% of TBM patients were classified as having treatment success defined as either cure or treatment completed. These numbers are worrisome and can primarily be explained by the very high mortality rate observed in this group. World Health Organization has set targets of reducing TB mortality rates by 50% by 2015 relative to 1990, a goal that is unlikely to be met in Eastern Europe—a region where both the TB and HIV epidemics are still increasing [15–18].

We have previously reported marked regional differences in outcome following a TB diagnosis in general among HIV-positive patients in Europe and Argentina. Approximately 30% of the patients in Eastern Europe died within the first year after a TB diagnosis, which is 4-5-fold higher than in Western Europe, but also 3-fold higher than in other middle-income countries such as Argentina [11]. The present study adds to the original report by documenting that these regional differences were also present for the three TB entities separately—and not least so for those presenting with extrapulmonary involvement, namely, TBM and TBEP, which in our setting to a large extent both could be characterized as disseminated TB disease.

Fifty-four clinics and cohorts in the European Region and Argentina contributed with data to this large study which has enabled identification of 100 clinical cases of TBM coinfected with HIV and included data from Eastern Europe, a region from where data remain scarce. Although TBM is a relatively rare disease, the size of the study has allowed for comparisons of characteristics and patient outcomes as well as analyzing regional differences in outcome.

Our study has several limitations. First of all, this was a retrospective study with observational data. Secondly, as an intrinsic limitation to TBM studies, TBM is a difficult diagnosis to establish and, consistent with other studies, only 38.0% of TBM cases were based on positive findings in the CSF. Further, the present study did not collect data on CSF-leucocyte count, CSF protein, and CSF glucose, which would have provided data to support the TBM diagnosis in patients with negative cultures. Additionally, as relevant differential diagnoses, 33% of TBM patients were diagnosed with other opportunistic infections around time of the TB diagnosis, including brain toxoplasmosis, progressive multifocal leucoencephalopathy and AIDS dementia. Finally, we cannot rule out that the loss of follow-up rate could have impacted the results, as also discussed previously [11]. Sensitivity analyses limited to the patients with a definite TB diagnosis and including only the first 12 months of follow-up showed consistent results with the main findings.

A large prospective observational cohort study of HIV-positive patients presenting with TB is currently recruiting patients in Europe and Latin America to help our understanding of how to improve the outcome for HIV- and TB-coinfected patients including those with most severe disease such as TBM [19].

## 5. Conclusion

In summary, HIV-positive patients with TBM in Europe and Argentina are characterized by severe immune suppression and by an alarmingly high mortality rate. The poor prognosis following TBM is especially true for patients from Eastern Europe and for patients with advanced HIV-infection, which urgently calls for public health interventions to improve patient management in terms of earlier diagnosis of HIV-infection, timely and accurate TB diagnosis, optimal TB treatment, and access to cART.

## Figures and Tables

**Table 1:** Baseline characteristics of HIV/TB coinfected patients according to the type of TB.

	TB meningitis		Pulmonary TB		Extrapulmonary TB		Chi-squared test*
	*N *	%	*N *	%	*N *	%	
All	100	(9.3)	371	(34.6)	601	(56.1)	
HIV characteristics							
Gender							
Male	74	(74.0)	234	(63.1)	422	(70.2)	0.028
Female	26	(26.0)	137	(36.9)	179	(29.8)	
Race							
White	75	(75.0)	224	(60.4)	377	(62.7)	0.026
Other	25	(25.0)	147	(39.6)	224	(37.3)	
Origin^1^							
Same country	75	(78.1)	300	(82.4)	436	(74.8)	0.0056
Other Europe	6	(6.3)	8	(2.2)	13	(2.2)	
Non-Europe	15	(15.6)	56	(15.4)	134	(23.0)	
HIV risk^2^							
IDU	55	(61.1)	183	(52.4)	279	(50.2)	0.17
Heterosexual	25	(27.8)	118	(33.8)	178	(32.0)	
Other	10	(11.1)	48	(13.8)	100	(17.9)	
Region							
Argentina	5	(5.0)	46	(12.4)	64	(10.7)	0.072
South	17	(17.0)	75	(20.2)	114	(19.0)	
Central	17	(17.0)	42	(11.3)	105	(17.5)	
East	61	(61.0)	208	(56.1)	318	(52.9)	
HBV							
Negative	33	(33.0)	117	(31.5)	243	(40.4)	0.027
Positive	5	(5.0)	31	(8.4)	51	(8.5)	
Unknown	62	(62.0)	223	(60.1)	307	(51.1)	
HCV							
Negative	11	(11.0)	58	(15.6)	109	(18.1)	0.044
Positive	35	(35.0)	106	(28.6)	210	(34.9)	
Unknown	54	(54.0)	207	(55.8)	282	(46.9)	
HIV before/at TB							
Yes	96	(96.0)	329	(88.7)	556	(92.5)	0.027
HIV at least 3 months before TB^3^	72	(75.0)	258	(78.4)	396	(71.2)	0.060
Prior AIDS^3^							
Yes	39	(40.6)	112	(34.0)	161	(29.0)	0.043
On cART^3^	16	(16.7)	74	(22.5)	87	(15.7)	0.035
Viral load^4^ (<400 cp/mL)	7	(21.9)	32	(26.9)	27	(13.8)	0.016

	Median	IQR	Median	IQR	Median	IQR	

Age (years)	33	28–40	33	28–41	33	28–39	0.37
HIV VL^4^ (Log_10_ cp/mL)	5.0	3.5–5.6	4.5	2.6–5.3	4.9	4.1–5.6	0.0075
CD4^5^ (/mm^3^)	113	40–269	236	101–492	143	58–304	<0.0001
Time HIV+ (months)	42	3–87	38	3–76	35	30–70	0.22
Date of TB diagnosis (month/year)	12/05	1/05–4/06	6/05	9/04–3/06	8/05	11/04–5/06	0.017
TB characteristics							
Prior TB^6^							
Yes	6	(6.7)	39	(11.5)	44	(7.7)	0.11
Disseminated TB	77	(77.0)	—	—	515	(85.7)	0.038**
Certainty of TB diagnosis							
Definite	46^12^	(46.0)	244	(65.8)	358	(59.6)	0.0037
Probable	24	(24.0)	43	(11.6)	96	(16.0)	
Presumptive	30	(30.0)	84	(22.6)	147	(24.5)	
Symptoms							
Yes	99	(99.0)	345	(93.0)	577	(96.0)	0.018
Fever	81	(81.8)	269	(78.6)	490	(84.8)	0.033
Weight loss	51	(51.5)	177	(51.3)	342	(59.3)	0.041
Cough with expectorate	26	(26.3)	229	(66.4)	201	(34.8)	<0.0001
Dry cough	12	(12.1)	59	(17.1)	132	(22.9)	0.013
Other	79	(79.8)	161	(46.7)	357	(61.9)	<0.0001
Anti-TB drug resistance							
H-resistant^7^							
Yes	7	(33.3)	24	(25.0)	18	(13.0)	0.016
Multidrug resistant^7^							
Yes	5	(23.8)	14	(14.7)	8	(5.8)	0.011
Initial anti-TB treatment							
RHZ	51	(51.0)	246	(66.3)	367	(61.1)	0.016
On 1st line drugs only^8^	35	(35.0)	209	(56.3)	307	(51.1)	0.0007
On 2nd line drug (s)^9^	65	(65.0)	162	(43.7)	294	(48.9)	

	Median	IQR	Median	IQR	Median	IQR	

Time since prior TB^10^ (months)	123	72–153	46	32–71	93	30–141	0.017
Weight^11^ (kg)	62	52–68	61	53–69	60	52–72	0.95

^1^Data available for 1043 (97.3%) at baseline. ^2^996 (92.9%) at baseline. ^3^Of 981 HIV positive at baseline. ^4^347 patients (35.4%) of those HIV positive at baseline. ^5^788 (80.3%) of those HIV+ at baseline. ^6^Of those where information about prior TB diagnosis is known, *N* = 1001. ^7^Data on resistance available for 256 patients. ^8^Ethambutol or streptomycin plus RHZ. ^9^Any other TB drug. ^10^Of those with a previous TB diagnosis. ^11^Of  703 (65.6%) with weight measured within 1 year of TB diagnosis. ^12^ *Mtb* documented in cerebrospinal fluid (CSF) for 38 patients—microscopy (24), PCR (7), and/or culture (17). H: isoniazid, R: rifamycin, and Z: pyrazinamide.

Baseline was defined as the date of TB diagnosis.

*Global *P* values comparing all three disease groups (TBM, TBP, and TBEP).

**The *P* value compares TBM and TBEP (by definition TBP cannot be disseminated).

IDU: injection drug user, HBV: hepatitis B, HCV: hepatitis C, HIV before/at TB: HIV diagnosed before TB diagnosis, HIV at least 3 months before TB: HIV diagnosed at least 3 months before TB diagnosis, VL: viral load, and time HIV+: number of months the patients have been HIV+ (Median).

**Table 2:** Multivariate poisson regression models for death in 3 separate TB groups.

		TB meningitis			Pulmonary TB			Extrapulmonary TB	
	aIRR**	95% CI	*P*	aIRR	95% CI	*P*	aIRR	95% CI	*P*
Region									
East versus other	5.41	2.58–11.34	<0.0001	2.87	1.62–5.08	0.0003	11.32	7.35–17.44	<0.0001
On cART at TB									
Yes versus no	0.25	0.11–0.58	0.0013	0.38	0.17–0.82	0.014	0.38	0.21–0.69	0.0016
Prior AIDS at TB									
Yes versus no	4.82	2.61–8.92	<0.0001	0.92	0.57 – 1.49	0.74	1.55	1.07–2.25	0.022
Multidrug resistant									
Yes versus no	0.30	0.06–1.43	0.13	1.72	0.75–3.94	0.20	0.71	0.22–2.29	0.57
Rifamycin in initial regimen									
Yes versus no	1.18	0.62–2.23	0.62	0.81	0.48–1.35	0.42	0.85	0.61–1.18	0.32
Disseminated									
Yes versus no	2.12	0.88–5.13	0.095	∗	∗	∗	1.42	0.83–2.42	0.20
CD4 at TB									
≤200 cells/mm^3^	1.00	0.52–1.92	0.99	1.99	1.15–3.43	0.014	2.40	1.65–3.50	<0.0001
>200 cells/mm^3^	1.00	—	—	1.00	—	—	1.00	—	—
Unknown	0.54	0.23–1.20	0.17	1.27	0.78–2.08	0.34	1.90	1.27–2.84	0.0019
Age									
Per 10 yr older	0.81	0.60–1.10	0.18	0.89	0.68–1.15	0.37	1.07	0.90–1.27	0.24
Diagnosis									
Definitive versus presumptive/probable	1.33	0.74–2.37	0.34	0.86	0.54–1.37	0.53	0.72	0.53–0.97	0.033

*No patients with disseminated disease in pulmonary TB group.

**The adjusted incidence rate ratios (aIRRs) were adjusted for all other variables in the model.

Tests for interaction: TB type *region (*P* = 0.0004), TB type *on cART at TB (*P* = 0.90), TB type *AIDS (*P* = 0.0003), TB type *MDR (*P* = 0.28), TB type *rifamycin in regimen (*P* = 0.13), TB type *CD4 (*P* = 0.028), TB type *age (*P* = 0.73), TB type *diagnosis type (*P* = 0.74), and TB type *disseminated disease (*P* = 0.83).
